# Supplementary material for: Identification of photoperiod-induced specific miRNAs in the adrenal glands of Sunite sheep (Ovis aries)
Source: Front Vet Sci. 2022 Jul 22;9:888207. doi: 10.3389/fvets.2022.888207 (PMC9354845; doi:10.3389/fvets.2022.888207)
Supplement: Supplementary file 1 [file Data_Sheet_1.ZIP › Supplementary file/miRDeep2 file.zip!/miRDeep2 file/Know_Structure/SP42A3/oar-mir-485.pdf]

miRBase precursor : oar-mir-485
Total read count : 879
oar-miR-485-5p read count : 287
oar-miR-485-3p read count : 592
remaining reads : 0

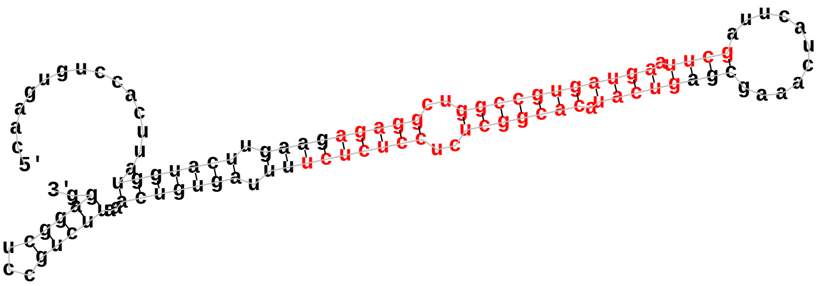

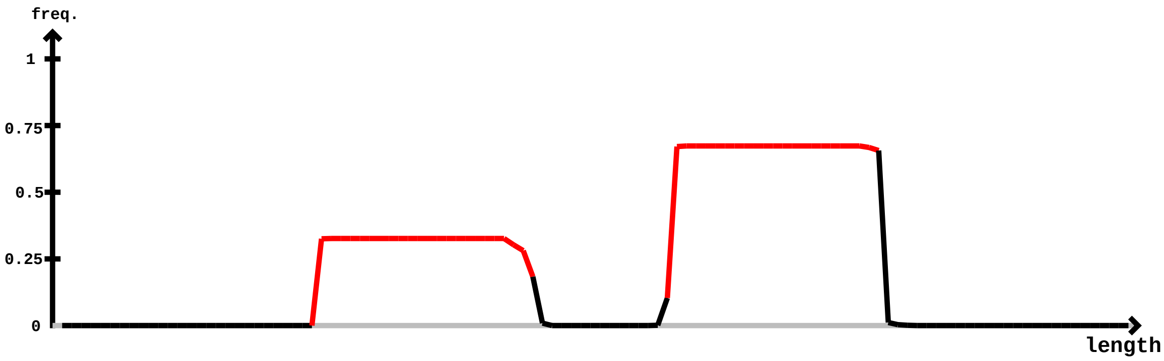

oar-miR-485-5p

oar-miR-485-3p

```
5'- caaguguccacuuaugguacuugaagagaggcuggccgugaugaauucgauucaucaaagcgagucauacacggcucuccucucuuuuagugucaaauucugccucggagg   -3'    exp
    ((((((.(((....)))))))))((((((((..(((((((((((.((((...........)))))))).)))))))..))))))))...........(((((...)))))).    reads    mm    sample
    ..........................agaggcuggccgugaugaau.................................................................    21       0     seq
    ..........................agaggcuggccgugaugaauu................................................................    19       0     seq
    ..........................agaggcuggccgugaugaauuc...............................................................    85       0     seq
    ..........................agaggcuggccgugaugaauucg..............................................................    153      0     seq
    ..........................agaggcuggccgugaugaauucga.............................................................    8        0     seq
    ...........................gaggcuggccgugaugaauuc...............................................................    1        0     seq
    .............................................................gagucauacacggcucuccucu............................    1        0     seq
    ..............................................................agucauacacggcucuccucu............................    1        0     seq
    ..............................................................agucauacacggcucuccucuc...........................    1        0     seq
    ..............................................................agucauacacggcucuccucucu..........................    88       0     seq
    ...............................................................gucauacacggcucuccucu............................    3        0     seq
    ...............................................................gucauacacggcucuccucuc...........................    9        0     seq
    ...............................................................gucauacacggcucuccucucu..........................    477      0     seq
    ...............................................................gucauacacggcucuccucucuu.........................    7        0     seq
    ...............................................................gucauacacggcucuccucucuuu........................    2        0     seq
    ...............................................................gucauacacggcucuccucucuuuu.......................    1        0     seq
    ................................................................ucauacacggcucuccucucu..........................    2        0     seq
```
